# Attitudes toward animals, and how species and purpose affect animal research justifiability, among undergraduate students and faculty

**DOI:** 10.1371/journal.pone.0233204

**Published:** 2020-05-29

**Authors:** Eric P. Sandgren, Robert Streiffer, Jennifer Dykema, Nadia Assad, Jackson Moberg

**Affiliations:** 1 Pathobiololgical Sciences, School of Veterinary Medicine, University of Wisconsin-Madison, Madison, WI, United States of America; 2 Medical History and Bioethics, School of Medicine and Public Health, University of Wisconsin-Madison, Madison, WI, United States of America; 3 University of Wisconsin-Madison Survey Center, University of Wisconsin-Madison, Madison, WI, United States of America; University of Lincoln, UNITED KINGDOM

## Abstract

As members of a university community that sponsors animal research, we developed a survey to improve our knowledge about factors underlying the perceived justifiability of animal research among faculty and undergraduate students. To accomplish this objective, we gathered quantitative data about their general views on animal use by humans, their specific views about the use of different species to address different categories of scientific questions, and their confidence in the translatability of animal research to humans. Students and faculty did not differ in their reported levels of concern for the human use of animals, but women reported significantly higher levels of concern than men. Among students, experience with animal research was positively correlated with less concern with animal use, and having practiced vegetarianism or veganism was associated with more concern. Gender, experience with animal research, and dietary preferences were similarly correlated with the extent of justifiability of animal use across all research purposes and species. Faculty responses resembled those for students, with the exception that justifiability varied significantly based on academic discipline: biological sciences faculty were least concerned about human use of animals and most supportive of animal research regardless of purpose or species. For both students and faculty, justifiability varied depending on research purpose or animal species. Research purposes, ranked in order of justifiability from high to low, was animal disease, human disease, basic research, human medicine, animal production, chemical testing, and cosmetics. Justifiability by purpose was slightly lower for students than for faculty. Species justifiability for students, from high to low, was small fish, rats or mice, pigs or sheep, monkeys, and dogs or cats. Faculty order was the same except that monkeys and dogs or cats were reversed in order. Finally, confidence in the translatability of animal research to our understanding of human biology and medicine was not different between students and faculty or between genders, but among faculty it was highest in biological sciences followed by physical sciences, social sciences, and then arts and humanities. Those with experience in animal research displayed the most confidence, and vegetarians/vegans displayed the least. These findings demonstrate that, although the range of views in any subcategory is large, views about animal research justifiability can vary significantly among respondent subpopulations in predictable ways. In particular, research purpose and choice of animal species are important variables for many people. This supports the claim that ensuring purpose and species are robustly integrated into research proposal reviews and approvals should be considered to be a best practice. We suggest that strengthening this integration beyond what is described in current regulations would better meet the justifiability criteria expressed by members of our campus community.

## Introduction

The bonds between humans and animals were established through thousands of years of cohabitation and evolving mutual dependency. The consequences of these connections, though, are complicated and often inconsistent [1]. Our contradictory relationships with animals are manifested in our views and practices regarding the use of animals in research. Animal research has long contributed to major scientific and medical advances, but often at significant costs to the lives and wellbeing of the research animals themselves. Because people care about animals, our society has developed a system of animal research oversight and regulation that, currently, includes evaluation of specific animal research proposals by considering the potential benefits to humans or other animals as well as the corresponding animal harms [2–6].

The development of social policy related to animal research requires information about the public’s attitudes toward animals and the use of animals to benefit humans or other animals. Population-based experiences and attitudes about animals can be gathered using survey methods [7]. One measure of human attitudes toward animal use is the 20-item Animal Attitude Scale (AAS), developed by Herzog and colleagues [8], which measures “general attitudes toward the human use of other species” [9]. Shorter scales, based on the AAS-20, have been developed and validated to measure attitudes toward animal protection and various kinds of human-animal interactions [9].

The overall objective in the current study was to identify attitudes toward the use of animals in research among undergraduate students in baccalaureate programs and faculty in all disciplines. For members of our university community, we sought to investigate general views toward animals and their views specifically regarding the use of particular species in research designed to address specific types of scientific questions. We aimed to create and validate a scale to capture a subject’s support for or opposition to animal research, and to describe how their support relates to species used, research purpose, and beliefs about translatability. This information can be used both to inform campus discussions and to direct and legitimize local and national policy-making about this important issue [10,11].

To achieve these objectives, we posed four research questions. First, what are people’s general views in support of or opposition to the use of animals by humans? To address this question, we administered the AAS-6, a six-item sub-scale slightly modified from Herzog’s AAS AAS-20. Second, how does research purpose influence attitudes on animal research justifiability? Third, how does the species used influence attitudes on animal research justifiability? To address these two questions, we created two new scales. Fourth, how translatable do people think animal research is to humans? To address this question, we asked respondents about the extent to which they thought the results of research studies using animals help people to learn more about humans.

Our survey was administered to randomly selected undergraduate students in each year of study and to all faculty at the University of Wisconsin-Madison (UW-Madison). Survey questions also asked for self-reports about the importance of and knowledge about animal research and its regulation, trustworthiness of sources of information about the topic, the frequency with which those resources were utilized, and whether respondents wanted more or less information from each [12]. In this report, we describe respondent’s views on animals, and how those views, the specific research objectives, and the species employed correlate with attitudes toward justifiability and translatability of animal research. Collectively, our findings help us to understand our university community’s current position on animal research and the elements that form the basis of those positions. More importantly, they provide guidance on how we might collectively refine the review process for animal research in the future.

## Materials and methods

We conducted a web survey to gauge student and faculty attitudes and beliefs about animal research. In the fall of 2016 at the University of Wisconsin-Madison, 2,000 undergraduate students were randomly selected from each year (e.g., freshman, sophomore, junior, and senior, measured by credits completed) for a total sample of 8,000 (out of 29,536 enrolled). Students were contacted by an email invitation sent by the University of Wisconsin Survey Center (UWSC) with the subject line “UW-Madison Wants Your Thoughts on Animal Research!”, which included an embedded URL with student’s username and password [13]. Nonresponding students received three email reminders. In total, 782 students completed the questionnaire for a response rate of 9.8% [14], which raised the concern that respondents may have been drawn from subsets of the population with either more or less favorable views about animal research. However, although our response rate was low, the high number of responses from our student population should provide reliable estimates: Fosnacht and colleagues [15] reported that “[w]ith few exceptions, we found estimates for several measures of college student engagement to be reliable under low response rate conditions (5%-10%), provided the sampling frame included at least 500 students”. Unlike many student surveys on attitudes towards animals, our student population was not limited to psychology majors or those enrolled in a psychology course, but instead included students across a full range of subjects. Additional survey characteristics have been described [12]. Finally, the survey questions were developed with input from diverse perspectives about animal research, including scientists, veterinarians, and animal activists. All aspects of this study were approved by the UW-Madison Education and Social/Behavioral Science Institutional Review Board.

In addition, a census of the 2,153 University of Wisconsin-Madison faculty members was undertaken in the spring of 2017. All faculty members were invited to participate using a postal letter that included a URL and authentication credentials to access the survey instrument. The letter also included a $2-bill as an incentive. Nonresponding faculty members received up to three emailed reminders and a final paper copy of the questionnaire in a postal mailing. In total, 942 faculty members completed the questionnaire for a response rate of 44% [14].

Students were asked for their year in school and current or anticipated major(s), which we used to assign student academic discipline. Faculty were asked for academic rank, and data on faculty discipline was provided by the university. Remaining questions were identical. Demographic questions asked for gender, year of birth, and past experiences with animals. Respondents were not asked to identify their political ideology. Though our findings may reflect views present at other large research schools/colleges, to the extent that academic demographics vary from those of the rest of society, we should be cautious about extending our findings to other populations.

The statistical analyses were conducted with STATA (Stata Corporation) using non-parametric tests, which do not assume a normal distribution of the dependent variables. Bivariate analyses completed included Wilcoxon/Mann-Whitney tests and Kruskal-Wallis tests. For categorical dependent variables, we used an ordinal logistic regression model for our estimation with a proportional odds assumption of identical log-odds ratio across categories. For dichotomous dependent variables, we used logistic regression for estimation. Significance is indicated with the following notation: * p < 0.05; ** p < 0.01; *** p < 0.001.

## Results

Respondent characteristics are presented in Table 1.

**Table 1:** Categories and numbers of survey participants^1^.

Participant category	Student, n %	Faculty, n%
**All, n**	782	942
**Demographics %**		
Gender		
Male	36.7	65.8
Female	61.6	31.1
Discipline		
Biological Science	44.5	38.4
Physical Science	20.2	19.6
Social Science	22.5	24.2
Arts and Humanities	7.2	17.8
Year in school		
Freshman	29.7	n/a
Sophomore	22.0	n/a
Junior	24.9	n/a
Senior	23.4	n/a
Faculty rank		
Assistant Professor	n/a	20.4
Associate Professor	n/a	19.4
Full Professor	n/a	60.2
**Experiences with animals %**		
Dietary preferences, last 5 years		
Vegetarian/vegan	19.6	16.6
Not vegetarian/vegan	80.4	83.4
Experience with animal research		
Worked on animal res. project	14.0	29.8
Not worked on an. res. project	86.0	70.2
**Support for animal research %**: “I do not think that there is anything wrong with using animals in medical research.”^2^		
Agree	43.4	60.6
Neither agree nor disagree	21.7	18.6
Disagree	35.0	20.9

^1^From [12]. Some respondents did not answer every survey question, yet were retained for remaining data analysis, so subcategories do not always add up to their population total.

^2^We scored answers to AAS-6 question [b] (here called question IVA; see Table 2) as “agree” (pooled strongly agree and agree), “neither agree nor disagree”, or “disagree” (pooled strongly disagree and disagree).

### Attitude scales

We asked a series of questions (Table 2) to measure (i) respondents’ general views on the use of animals by humans using the animal attitudes scale (AAS-6) [8,9]; (ii) attitudes toward specific animal research objectives using the “purpose” subscale of the animal research attitudes scale (ARAS-P); and (iii) the influence of animal species on perceived justifiability of animal research using the “species” subscale of the animal research attitudes scale (ARAS-S). For each respondent, the order of items within each scale was randomized. As discussed later, past research indicates that gender and people’s experiences with animals (including dietary choices and animal research participation) influence their views on animals. Thus, we assessed concurrent validity of our scales by examining the relationship between scale scores and each these variables. Next, we asked about respondents’ belief in translatability of animal research to human biology and medicine (question V). Finally, as a measure of respondents’ preexisting support for animal research, we scored answers to AAS-6 question [b] (here labeled as question IVA; see Table 2) as “agree” (pooled strongly agree and agree), “neither agree nor disagree”, or “disagree” (pooled strongly disagree and disagree).

**Table 2:** Survey questions.

Question	Question wording	Responses
AAS-6	To what extent do you agree or disagree with each of the following statements about the use of animals? [a] It is morally wrong to hunt wild animals for sport; “Hunt” ‡ [b] I do not think that there is anything wrong with using animals in medical research; “Research” [c] I think it is perfectly acceptable for cattle and hogs to be raised for human consumption; “Consumption” [d] It is unethical to breed purebred dogs when millions of dogs are killed in animal shelters each year; “Breed” ‡ [e] I sometimes get upset when I see wild animals in cages at zoos; “Zoos” ‡ [f] Basically, humans have the right to use animals as we see fit; “Right to use”	1 = Strongly agree; 2 = Agree; 3 = Neither agree nor disagree; 4 = Disagree; 5 = Strongly disagree
ARAS-P	How often do you feel it is justifiable to use animals in research studies for each of the following purposes? [a] To look for ways to prevent or treat animal diseases; “Anim. Dis.” [b] To improve the production of livestock to lower the cost or raise the quality of agricultural products such as meat, milk, and eggs; “Anim. Prod.” [c] To conduct basic research to learn more about how organs, tissues, and cells function; “Basic Res.” [d] To look for ways to prevent or treat human diseases; “Hum. Dis.” [e] To test new medications for humans; “Hum. Med.” [f] To test the safety of workplace or household chemicals; “House Chem.” [g] To test the safety of cosmetics; “Cosmetics”	1 = Never; 2 = Rarely; 3 = Sometimes; 4 = Usually; 5 = Always
ARAS-S	How often do you feel it is justifiable to use each of the following types of animals in research studies? [a] Monkeys [b] Dogs and cats [c] Pigs and sheep [d] Rats and mice [e] Small fish such as minnows or zebrafish	1 = Never; 2 = Rarely; 3 = Sometimes; 4 = Usually; 5 = Always
IVB	In the past 5 years, have you ever… [a] been a vegetarian or vegan? [b] worked on a research project that used animals?	1 = Yes; 2 = No
V	To what extent do you think that results of research studies using animals help people to learn more about humans?	1 = Not at all; 2 = A little; 3 = Somewhat; 4 = Quite a bit; 5 = A great deal
IVA	To what extent do you agree or disagree with the following statement about the use of animals? I do not think that there is anything wrong with using animals in medical research.	1 = Strongly agree; 2 = Agree; 3 = Neither agree nor disagree; 4 = Disagree; 5 = Strongly disagree

‡Reverse scored in AAS6; see Table 3 footnote. Our 1–5 scale in the survey questions also reversed the order of agreement relative to Herzog and colleagues.

### Characteristics and validation of AAS-6, ARAS-P, and ARAS-S

As noted previously, surveys based on the AAS-20 measure people’s attitudes about human use of animals. The original AAS had 20 questions (AAS-20), and shortened versions with five questions (AAS-5) or 10 questions (AAS-10) also were developed and validated [9]. All six questions chosen for our AAS-6 were in the AAS-10, and four of our questions also were present in the AAS-5. Compared to AAS-5, we selected a sixth question in order to have three worded in favor of animal use and three worded in opposition to animal research. We also substituted “It is unethical to breed purebred dogs when millions of dogs are killed in animal shelters each year” for “The slaughter of whales and dolphins should be immediately stopped even if it means some people will be put out of work” because the former seemed less one-sided.

Thus, our AAS-6, adapted from Herzog’s work, asks for level of agreement or disagreement with six questions, three for which agreement (lower scores) indicates a more protective attitude toward animals (marked with ‡ in Table 3), and three for which the opposite is true. For the question on AAS-6 about hunting, student and faculty scores did not differ. Faculty indicated more agreement with human use of animals for all other purposes except the human right to use animals as they see fit (Table 3; Wilcoxon/Mann-Whitney test). Principal component analysis (PCA) is a method for conducting exploratory factor analysis and to verify unidimensionality. PCA suggested a one-component solution for AAS-6 (only the first component had an eigenvalue >1). The first component explained 51% or 43% of the variance, respectively, for students and faculty, with remaining variance unaccounted for (Table 3). Cronbach’s Alpha, a measure of the internal consistency or interrelatedness among items in a scale, was either 0.81 or 0.73, indicating good reliability. To create the composite scale, the scores marked with ‡ are reversed, and then all scores are summed so that higher AAS-6 scores indicate stronger protective attitudes toward animals. We then divided by six, the number of questions, to normalize the composite score to a 1–5 scale. Mean scale scores are presented in Table 4.

**Table 3:** Mean level of agreement for items in AAS-6 (1–5 scale).

Question	Student Scores X (SD)	Faculty Scores X (SD)	Stu. vs. Fac.^1^p
Hunt‡	2.9 (1.3)	2.9 (1.3)	0.72 ns
Research	2.9 (1.2)	2.4 (1.2)	<0.000***
Consumption	2.3 (1.2)	2.1 (1.1)	0.001***
Breed‡	2.7 (1.1)	3.1 (1.1)	<0.000***
Zoos‡	2.4 (1.2)	2.5 (1.0)	0.016*
Right to use	3.9 (1.1)	4.1 (1.0)	0.003**
PCA component 1 portion of variance	0.51	0.43	
Cronbach’s Alpha	0.81	0.73	

ns, not significant

*p<0.05

**p<0.01

***p<0.001.

^1^Wilcoxon/Mann-Whitney test.

‡For these three questions in AAS6, lower scores (rather than higher scores as is the case for the other three questions) represent a more positive attitude toward animal wellbeing issues (or a less positive attitude toward human use of animals). For that reason, values for the three marked questions were reverse coded when creating the AAS-6 so that higher scores in the composite value (see Table 3) would indicate more positive attitudes toward animal wellbeing.

**Table 4:** AAS-6 multivariate analyses (1–5 scale).

Respondent characteristics	Students					Faculty					Students vs. faculty		
	Mean	SD	Odds Ratio P		[95% CI]	Mean	SD	Odds Ratio P		[95% CI]	Odds Ratio P		[95% CI]
All	3.2	.84				3.0	.73						
Students vs (Faculty)											1.5	.206	[.80, 2.9]
Gender													
(Male)	2.8	.82				2.9	.69						
Female	3.4	.75	2.7	.000	[1.7, 4.2]	3.3	.69	2.3	.000	[1.5, 3.5]	2.1	.000	[1.5, 3.0]
Division													
(Biological Sciences)	3.2	.78				2.8	.69						
Physical Sciences	2.9	.83	.89	.650	[.54, 1.5]	2.9	.67	1.5	.043	[1.0, 2.2]	1.4	.074	[.97, 2.0]
Social Sciences	3.3	.87	1.2	.518	[.66, 2.3]	3.1	.71	1.7	.019	[1.1, 2.6]	1.7	.011	[1.1, 2.5]
Humanities	3.3	1.0	.80	.629	[.33, 2.0]	3.4	.71	5.0	.000	[3.1, 8.1]	3.9	.000	[2.5, 6.1]
Year in School													
(Freshman)	3.2	.84											
Sophomore	3.1	.80	.81	.255	[.56, 1.2]								
Junior	3.1	.92	.69	.042	[.49, .99]								
Senior	3.2	.78	.87	.452	[.61, 1.2]								
Academic Rank													
(Assistant Professor)						3.2	.74						
Associate Professor						3.1	.73	.96	.846	[.67, 1.4]			
Full Professor						2.9	.70	.67	.007	[.50, .89]			
Worked on Animal Research													
(Yes)	2.9	.78				2.7	.67						
No	3.2	.84	1.8	.002	[1.2, 2.6]	3.1	.72	1.3	.099	[.95, 1.8]	1.3	.136	[.93, 1.8]
Vegetarian or vegan													
(Yes)	3.9	.80				3.6	.75						
No	3.0	.75	.12	.000	[.08, .17]	2.9	.65	.18	.000	[.13, .25]	.19	.000	[.14, .26]
Student vs Faculty Interactions													
Student X Female											1.5	.057	[1.1, 2.3]
Student X Physical Sciences											.65	.115	[.39, 1.1]
Student X Social Sciences											.64	.093	[.38, 1.1]
Student X Humanities											.45	.020	[.23, .88]
Student X No An. Res.											1.5	.103	[.92, 2.4]
Student X Not Veg. or Vegan											.60	.028	[.38, .95]
Model fit statistics													
N			738					935			1673		
Pseudo R2			.0545					.0544			.0543		
Log likelihood			-2089					-2533			-4652		

The ARAS-P provides a list of seven objectives or purposes for which one might experiment on animals, and the ARAS-S provides a list of five categories of experimental animal species. They ask respondents to evaluate the justifiability of each purpose or species in the context of research. Unlike the AAS, higher scores on these two scales indicate greater justifiability (or less protective views toward animals). Individual scores are summed, and, once again, we divided total scores by the number of questions in each scale so that each final ARAS-P or ARAS-S score would be expressed on a 1 to 5 scale. Individual purpose and species scores are presented in Table 5. Composite scores are presented in Tables 6 and 7. For both ARAS-P and ARAS-S, PCAs indicated one component solutions, and Cronbach’s alphas indicated good reliability (Table 5).

**Table 5:** Responses underlying ARAS-P and ARAS-S (1–5 scales)^1^.

Scale	Student	Faculty	Student vs. Faculty^2^
	X (SD)	X (SD)	p
ARAS-P			
Animal disease	4.1 (0.9)	4.2 (0.8)	0.047*
Human disease	3.8 (1.1)	4.1 (0.8)	<0.000***
Basic research	3.7 (1.0)	4.0 (0.9)	<0.000***
Human medicine	3.4 (1.2)	3.9 (0.9)	<0.000***
Animal production	3.3 (1.1)	3.6 (1.0)	<0.000***
Chemicals	2.6 (1.2)	3.2 (1.1)	<0.000***
Cosmetics	2.1 (1.1)	2.4 (1.1)	<0.000***
PCA	0.66	0.67	
Cronbach’s Alpha	0.91	0.91	
ARAS-S			
Small fish	3.7 (1.1)	4.0 (0.9)	<0.000***
Rat, mouse	3.7 (1.1)	3.9 (0.9)	0.038*
Pig, sheep	3.0 (1.2)	3.4 (1.0)	<0.000***
Monkeys	2.7 (1.2)	2.9 (1.0)	<0.000***
Dog, cat	2.5 (1.2)	3.1 (1.0)	<0.000***
PCA	0.76	0.74	
Cronbach’s Alpha	0.92	0.91	

^1^Purpose and species listed in order of decreasing justifiability for students

^2^Wilcoxon/Mann-Whitney test

**Table 6:** ARAS-P multivariate analysis (1–5 scale).

Respondent characteristics	Students					Faculty					Faculty vs. Students		
	Mean	SD	Odds Ratio P		[95% CI]	Mean	SD	Odds Ratio P		[95% CI]	Odds Ratio P		[95% CI]
All	3.3	.89				3.6	.76						
Students vs (Faculty)											.52	.041	[.28, .97]
Gender													
(Male)	3.6	.77				3.6	.73						
Female	3.1	.87	.50	.001	[.33, 76]	3.4	.78	.67	.060	[.44, 1.0]	.74	.102	[.52, 1.1]
Division													
(Biological Sciences)	3.3	.82				3.9	.66						
Physical Sciences	3.5	.85	1.2	.373	[.77, 2.0]	3.6	.70	.62	.017	[.42, .92]	.66	.023	[.46, .94]
Social Sciences	3.1	.93	.88	.668	[.49, 1.6]	3.4	.72	.48	.001	[.31, .73]	.53	.002	[.36, .79]
Humanities	3.1	1.0	1.3	.578	[.54, 3.0]	3.2	.79	.25	.000	[.15, .39]	.29	.000	[.19, .46]
Year in School													
(Freshman)	3.3	.88											
Sophomore	3.3	.90	1.0	.982	[.69, 1.4]								
Junior	3.3	.89	1.1	.589	[.78, 1.6]								
Senior	3.3	.89	1.3	.127	[.92, 1.9]								
Academic Rank													
(Assistant Professor)						3.4	.75						
Associate Professor						3.6	.81	1.3	.155	[.90, 1.9]			
Full Professor						3.7	.74	1.6	.002	[1.2, 2.1]			
Worked on Animal Research													
(Yes)	3.6	.85				4.0	.65						
No	3.2	.88	.44	.000	[.31, .63]	3.5	.75	.51	.000	[.37, .71]	.53	.000	[.38, .73]
Vegetarian or vegan													
(Yes)	2.7	.93				3.1	.92						
No	3.4	.81	4.3	.000	[3.1, 6.2]	3.7	.69	3.1	.000	[2.2, 4.3]	3.0	.000	[2.2, 4.2]
Student vs Faculty Interactions													
Student X Female											.54	.002	[.37, .76]
Student X Physical Sciences											1.8	.028	[1.1, 3.0]
Student X Social Sciences											1.3	.246	[.81, 2.2]
Student X Humanities											2.4	.011	[1.2, 4.6]
Student X No An. Res.											.76	.271	[.47, 1.2]
Student X Not Veg. or Vegan											1.5	.071	[.96, 2.4]
Model fit statistics													
N			738					934			1672		
Pseudo R2			.0380					.0406			.0431		
Log likelihood			-2244					-2684			-4961		

**Table 7:** ARAS-S multivariate analyses (1–5 scale).

	Students					Faculty					Students vs. Faculty		
Respondent characteristics	Mean	SD	Odds Ratio P		[95% CI]	Mean	SD	Odds Ratio P		[95% CI]	Odds Ratio P		[95% CI]
All	3.1	1.0				3.5	.83						
Students vs (Faculty)											.95	.863	[.50, 1.8]
Gender													
(Male)	3.6	.92				3.6	.78						
Female	2.9	.97	.49	.001	[.32, .75]	3.2	.85	.66	.044	[.43, .99]	.76	.126	[.54, 1.1]
Division													
(Biological Sciences)	3.2	.93				3.7	.72						
Physical Sciences	3.4	1.0	1.2	.382	[.76, 2.1]	3.5	.78	.80	.257	[.55, 1.2]	.88	.478	[.61, 1.3]
Social Sciences	2.9	1.0	.94	.850	[.52, 1.7]	3.3	.84	.65	.048	[.42, 1.2]	.71	.088	[.48, 1.1]
Humanities	2.9	1.1	1.4	.528	[.52, 3.6]	3.0	.86	.31	.000	[.19, .49]	.38	.000	[.25, .61]
Year in School													
(Freshman)	3.1	1.0											
Sophomore	3.1	1.0	.90	.575	[.62, 1.3]								
Junior	3.2	1.0	1.2	.356	[.83, 1.7]								
Senior	3.3	.98	1.6	.007	[1.1, 2.3]								
Academic Rank													
(Assistant Professor)						3.3	.87						
Associate Professor						3.4	.85	1.0	.959	[.70, 1.5]			
Full Professor						3.5	.80	1.3	.130	[.94, 1.7]			
Worked on Animal Research													
(Yes)	3.6	.89				3.8	.73						
No	3.1	1.0	.42	.000	[.29, .60]	3.3	.94	.64	.007	[.46, .89]	.65	.009	[.47, .90]
Vegetarian or vegan													
(Yes)	2.4	1.1				2.9	.97						
No	3.3	.91	4.6	.000	[3.3, 6.6]	3.6	.75	3.5	.000	[2.5, 4.9]	3.3	.000	[2.4, 4.5]
Interaction Terms (If Significant)													
Female X Physical Sciences			.42	.029	[.20, .92]			.88	.432	[.58, 2.3]	.55	.035	[.31, .96]
Female X Social Sciences			.60	.161	[.30, 1.2]			.72	.692	[.31, 1.6]	.75	.209	[.47, 1.2]
Female X Humanities			.28	.028	[.09, .87]			1.2	.662	[.58, 2.3]	.80	.449	[.45, 1.4]
Student vs Faculty Interaction Terms													
Student X Female											.48	.000	[.32, .71]
Student X Physical Sciences											1.2	.430	[.73, 2.1]
Student X Social Sciences											1.2	.547	[.70, 1.9]
Student X Humanities											1.7	.144	[.84, 3.3]
Student X No An. Res.											.57	.021	[.35, .92]
Student X Not Veg. or Vegan											1.5	.093	[.93, 2.4]
Model fit statistics													
N			738					932			1670		
Pseudo R2			.0497					.0374			.0458		
Log likelihood			-2012					-2403			-4446		

**Fig 1:**
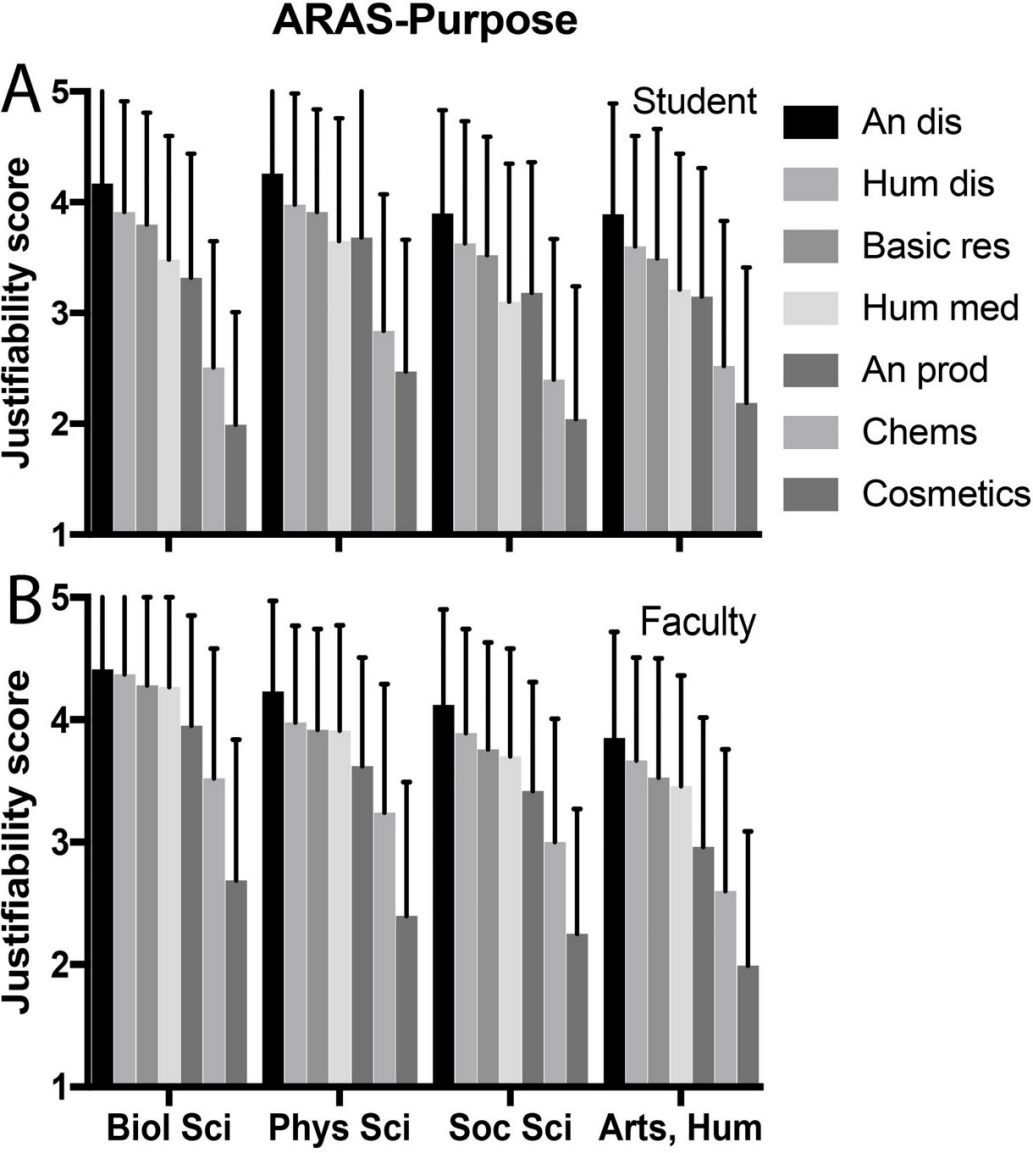
Purpose justification by discipline, in decreasing order of justifiability. (A) Students. (B) Faculty. Data presented as mean ± standard deviation.

**Fig 2:**
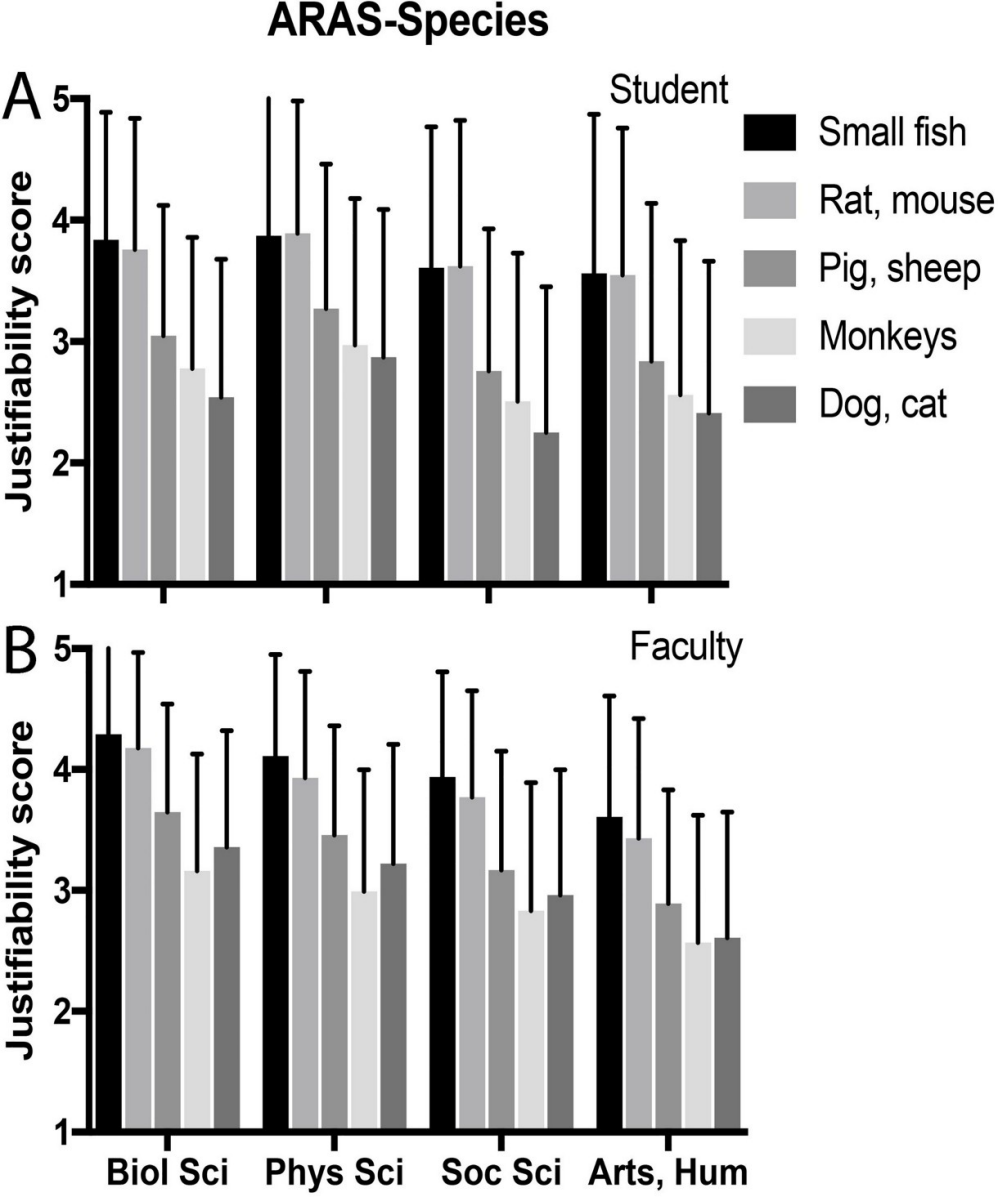
Species justification by discipline, in decreasing order of justifiability for students. (A) Students. (B) Faculty. Data presented as mean ± standard deviation.

For AAS-6, student and faculty scores were not significantly different by multivariate analysis (Table 4). For all objectives in ARAS-P and all species in ARAS-S, faculty scores indicated greater justifiability than students in bivariate analysis (Table 5), but after controlling for several confounding variables in multivariate analysis, student and faculty scores were only significantly different for the ARAS-P (Tables 6 and 7). Both groups identified the same order of justifiability for each objective, starting with the study of animal disease, followed in decreasing order by human disease, basic research, human medications, animal production, household chemicals, and cosmetics testing (Table 5, Fig 1). For ARAS-S, the species trend for students, from most to least justifiable, was small fish, rats and mice, pigs and sheep, monkeys, then dogs and cats (Table 5, Fig 2). For faculty, order was the same except that use of monkeys was least justifiable, and dogs and cats ranked just above monkeys. We found a high inverse correlation between AAS-6 and ARAS-P scores for students (r = 0.70; p< 0.001) and for faculty (r = 0.65; p<0.001). When comparing AAS-6 and ARAS-S, respective inverse correlations were (r = 0.73; p< 0.001) and (r = 0.65; p< 0.001). Finally, note that ARAS-S (species) scores are significantly lower than ARAS-P (purpose) scores; these can be compared quantitatively because the scoring criteria are the same for each scale.

Tables 4, 6 and 7 show that respondents’ AAS-6, ARAS-P, and ARAS-S scores generally vary based on gender and experiences with animals. In particular, women indicated a more positive attitude toward animal protection (higher AAS-6 scores), and a more negative attitude toward the use of animals across all research objectives and species (lower ARAS-P and ARAS-S scores). However, gender differences were significantly smaller for faculty than for students (p<0.05 for AAS-6 and p<0.001 for ARAS-P and ARAS-S), principally because female faculty scores were much closer to their male counterparts than was the case for students. In fact, for all scale scores, male students and male faculty did not differ significantly. Vegetarian/vegans scored higher on AAS-6 and lower on both ARAS scales compared to omnivores, and the opposite pattern was observed for individuals who had worked on an animal research project compared to those who had not.

For each scale we evaluated the influence on scores of academic discipline and duration on campus (Tables 4, 6 and 7, Figs 1 and 2). For AAS-6 among students, discipline did not have a significant effect on score after multivariate analysis when comparing each discipline to biology. Both social sciences and humanities were less likely to be supportive of animal use (higher scores) than physical sciences, but the differences were not significant when controlling for gender. Year in school showed only a slight difference among juniors. For faculty, disciplines were significantly different, with lowest to highest score in the order of biological science, physical science, social science, then arts and humanities (Fig 1). Among students, physical sciences displayed the highest scores for ARAS-P and ARAS-S, but multivariate analysis indicated that no disciplines were significantly different from the biological sciences. For faculty, all disciplines were significantly lower than biological sciences on both scales except physical sciences for ARAS-S. The faculty rank of full professor was associated with a slightly lower AAS-6 score and a slightly higher ARAS-P score.

We also compared student versus faculty discipline scores for each scale (Tables 4, 6 and 7). In general, for all scales, student and faculty scores different for biological sciences and social sciences, but minimally different for physical sciences and arts and humanities. Thus, overall student-faculty differences in scales can be attributed primarily to differences in biological and social sciences. For all scales, an interaction was present between student and female variables, consistent with our observation that the male-female difference in animal use approval was significantly greater among students than among faculty because of a change (decrease in AAS-6 and increases in ARAS-P and ARAS-S) in female faculty score compared to female student score. Another interaction was present between student and humanities variables for AAS-6 and ARAS-P, consistent with the lack of score difference between students and faculty in this discipline. This was also true for students and physical sciences variables for ARAS-P.

Finally, to evaluate how pre-existing support for animal research correlated with respondent scores on our scales, scores were compared based on response to the AAS-6 question b: “I do not think that there is anything wrong with using animals in medical research” (Table 8). There were striking positive correlations of ARAS-P and ARAS-S scores with the extent of support for animal research. We did not evaluate AAS-6 in this manner because the research support question was part of that scale.

**Table 8:** Purpose and species scales and confidence in animal research translatability versus response to survey question AAS-6b, “I do not think that there is anything wrong with using animals in medical research”.

Scale, response	Student Scores X (SD)	Faculty Scores X (SD)
ARAS-P		
Agree or strongly agree	3.9 (0.6)	4.0 (0.6)
Neither agree nor disagree	3.3 (0.6)	3.4 (0.5)
Disagree or strongly disagree	2.5 (0.8)	2.8 (0.8)
p^1^	368***	317***
ARAS-S		
Agree or strongly agree	3.9 (1.1)	3.8 (0.7)
Neither agree nor disagree	3.1 (0.6)	3.2 (0.5)
Disagree or strongly disagree	2.3 (0.8)	2.7 (0.8)
p^1^	392***	310***
Translatability		
Agree or strongly agree	4.5 (0.7)	4.6 (0.6)
Neither agree nor disagree	3.8 (0.9)	4.1 (0.8)
Disagree or strongly disagree	3.3 (1.0	3.8 (1.0)
p^1^	240***	155***

^1^Wilcoxon/Mann-Whitney test.

***p<0.001.

### Influences on the belief in translatability of animal research

To a large extent, animal research is conducted for the purpose of learning more about humans. As indicated in Table 9, confidence in translatability to humans of research findings using animals was generally high, close to or above “quite a bit” (a score of 4). Significant gender or student-faculty differences were not observed. Juniors and seniors expressed more confidence than freshmen, and associate and full professors expressed more confidence than assistant professors. Discipline differences were present only for faculty, with those in the biological sciences most confident, followed by physical sciences, social sciences, and then arts and humanities. Experience with animal research and being vegetarian/vegan were significant predictors of confidence in translatability, though in opposite directions, for both students and faculty. As for the purpose and species scales, an interaction between student and female was present. Finally, confidence in translatability of animal research was strongly correlated with pre-existing support for animal research (Table 8).

**Table 9:** Translatability (1–5 scale).

Respondent characteristics	Students					Faculty					Students vs. Faculty		
	Mean	SD	Odds Ratio P		[95% CI]	Mean	SD	Odds Ratio P		[95% CI]	Odds Ratio P		[95% CI]
All	3.9	1.0				4.3	.81						
Students vs (Faculty)											.82	.571	[.41, 1.6]
Gender													
(Male)	4.1	.90				4.4	.75						
Female	3.8	1.0	.66	.084	[.41, 1.1]	4.2	.89	.88	.616	[.52, 1.5]	1.0	.960	[.66, 1.5]
Division													
(Biological Sciences)	4.1	.92				4.6	.64						
Physical Sciences	4.0	1.0	.72	.233	[.42, 1.2]	4.2	.73	.46	.000	[.29, .70]	.50	.001	[.33, .75]
Social Sciences	3.6	1.0	.68	.241	[.36, 1.3]	4.1	.88	.50	.005	[.31, .82]	.55	.008	[.35, .85]
Humanities	3.8	1.0	.74	.539	[.29, 1.9]	3.9	.88	.28	.000	[.17, .48]	.31	.000	[.19, .50]
Year in School													
(Freshman)	3.8	1.0											
Sophomore	3.9	1.0	1.1	.568	[.76, 1.7]								
Junior	4.0	.92	1.6	.008	[1.1, 2.4]								
Senior	4.1	1.0	1.9	.001	[1.3, 2.8]								
Academic Rank													
(Assistant Professor)						4.2	.84						
Associate Professor						4.4	.71	1.5	.048	[1.0, 2.2]			
Full Professor						4.3	.82	1.4	.042	[1.0, 1.9]			
Worked on Animal Research													
(Yes)	4.4	.87				4.7	.62						
No	3.8	1.0	.35	.000	[.24, .53]	4.1	.84	.47	.000	[.32, .68]	.47	.000	[.32, .69]
Vegetarian or vegan													
(Yes)	3.6	1.1				4.0	.98						
No	4.0	.96	1.9	.001	[1.3, 2.6]	4.4	.76	1.7	.002	[1.2, 2.5]	1.7	.002	[1.2, 2.4]
Student vs Faculty Interactions													
Student X Female											.54	.005	[.35, .83]
Student X Physical Sciences											1.3	.378	[.73, 2.3]
Student X Social Sciences											1.0	.918	[.59, 1.8]
Student X Humanities											2.0	.055	[.99, 4.1]
Student X No An. Res.											.68	.172	[.39, 1.2]
Student X Not Veg. or Vegan											1.0	.880	[.64, 1.7]
Model fit statistics													
N			738					938			1676		
Pseudo R2			.0633					.0792			.0795		
Log likelihood			-901					-942			-1857		

## Discussion

In this report, we describe attitudes toward animal research among university undergraduates and faculty. Novel aspects of the study include: (1) presence of large respondent sample sizes, (2) data from students and faculty and at the same institution, (3) survey conducted over the full range of scientific disciplines, (4) assessment of views on translatability, (5) development and validation of two new research scales (for research purpose and species), and (6) and examination of correlations among the scales. Our findings allow us to address the four research questions we posed regarding perspectives on animal research justifiability by members of the University of Wisconsin-Madison community. Our first research question was “What are the respondents’ general views on the use of animals by humans?” We used the AAS-6 scale to address this question. In general, views identified in our study reflect those reported in other population studies [8,9]. Specifically, there was a strong gender difference, with women less supportive of use of animals by humans than men; less support of animal research by women compared to men has been reported in many other studies [8,16–26]. Henry and Pulcino [18] and Swami and colleagues [26] reported finding similar influences on attitudes about biomedical research using the Attitudes Toward the Treatment of Animals scale, and the Attitudes to Experimentation on Animals scale, respectively.

A comparison of scores across the multiple AAS versions can be obtained by dividing the total score by the number of questions in each AAS. Table 10 compares our AAS-6 with Herzog’s AAS-5 and AAS-10. Lower scores are associated with males compared to females in all studies. AAS-6 reproducibility, measured by Cronbach’s Alpha, approached that of AAS-5, though was lower than for the longer AAS-10. Per-question AAS scoring trends were consistent across scales. However, one should not compare the absolute magnitude of scores across survey versions that use different questions, given that AAS-5 and AAS-10 scores are quite different even though they were generated from the same data set. Also, Herzog’s study population consisted of adults recruited through the internet, so differs from ours [9].

**Table 10:** Comparison of AAS6, AAS5, and AAS10.

	Mean AAS score per question and statistical reliability^1^			
	AAS-6 student	AAS-6 faculty	AAS-5^2^	AAS-10^2^
Males	2.79	2.85	3.24	2.89
Females	3.39	3.30	3.86	3.43
Cronbach’s Alpha	0.81	0.73	0.82	0.90

^1^Scores normalized by summing individual question scores, then dividing by the number of questions in each scale.

^2^From ref. [9]

To further validate AAS-6, we compared scores for people reporting opposite animal experiences within the last five years. Categories included “vegetarian/vegan” and “worked on a research project with animals”. For each question about experiences, AAS-6 displayed the expected correlation of lower score with behaviors supportive of human use of animals, especially dietary history. Herzog’s AAS-5 and AAS-10 also demonstrated lower scores for people identifying as omnivores compared to those who did not eat meat [9].

Our second research question asked, “How does research purpose influence attitudes on animal research justifiability?” The animal research attitude scale-purpose (ARAS-P) posed seven questions about justifiability of animal use for specific research objectives, and the pattern of responses mirrored almost precisely that recorded for AAS-6, although higher scores on ARAS-P indicate greater justification of animal use. Order of justifiability after animal disease was human disease, basic research, testing human medicines, animal production, testing workplace and household chemicals, and testing cosmetics.

An intriguing finding among both study populations was that use of animals to look for ways to prevent or treat animal disease was believed to be more justifiable than the use of animals to look for ways to prevent or treat human disease or to test new medications for humans. Ipsos MORI surveys of the public in England also reported greater acceptance of research to understand animal health rather than human health [27]. This finding is in tension with the idea, often associated with the support of animal research, that the lives and welfare of humans are more important than the lives and welfare of the animals used in research to benefit others. It is, however, consistent with an ethical perspective in which cost to research participants most readily can be justified if the benefits accrue to the same population (species). The 2015 ruling by the U.S. Fish and Wildlife Service that listed chimpanzees as endangered species under the Endangered Species Act resulted in a similar, but stronger, priority of animals over humans, as the ruling only allows the harming or killing of chimpanzees for “scientific purposes that benefit the species in the wild, or to enhance the propagation or survival of chimpanzees, including habitat restoration and research on chimpanzees in the wild that contributes to improved management and recovery” [28]. A similar perspective seems to be reflected in the research regulations regarding (human) pediatric research, which allows Institutional Review Boards to approve a slightly increased level of risk in non-therapeutic pediatric research that is likely to provide information about a condition or disorder that the research subjects themselves have [29].

Most individuals disapproved of using animals to test cosmetics. The Ipsos MORI surveys also reported greater acceptance of medical research than testing of chemicals [27]. Other studies have addressed animal research acceptability as a function of experimental purpose, and identify rankings similar to ours, especially lower support for testing of household chemicals or cosmetics [18,24,30,31]; reviewed in [32]. For faculty, justifiability of the study of animal disease, basic research, human disease, and testing human medicines were only slightly different from one another, while for students they were more separated. Though basic research using animals has been criticized as being too distant from practical application, both students and faculty viewed this objective as a generally justifiable purpose for which to use animals. Men scored higher than women on justifiability for each purpose, and, in general, faculty justification for each purpose was higher than for students.

To address our third research question, “how does the species used influence attitudes on animal research justifiability?”, our animal research attitude scale-species (ARAS-S) asked about animal research justifiability for each of several example species. Judgments about species justifiability can be influenced by several characteristics of the species under consideration, particularly their status with respect to the human-animal bond, their position on the evolutionary phylogenetic scale, and the range of cognitive and emotive capacities they possess [1]. Dogs and cats, of course, are most closely associated with humans as companion animals, and to some individuals will be viewed as “part of the family”. For students, use of these species in research was least justifiable, followed by non-human primates. Non-human primates are phylogenetically most like humans and exhibit a tremendous range of cognitive and emotive capacities, and so their use in research is most difficult to justify ethically when we cannot justify using humans [33,34]. For faculty, use of these species was least justifiable, followed by dogs and cats. In both study populations, non-human primates and dogs and cats were followed by pigs and sheep, with the use of rodents and small fish being ranked most justifiable. These findings are consistent with the suggestion that justifiability is based on a combination of familiarity, phylogeny, and capacities, with different emphasis between students and faculty [30,35–37]. A similar pattern of unequal species-based acceptability of animal research has been reported in other studies as well [18,24,25,31,38–40]. As for purpose, men scored higher than women on justifiability for each species, but student-faculty differences were not significant after accounting for other variables.

Interestingly, among all demographic groups in our respondent populations, the species scores were lower than purpose scores, even though both used the same five-point justifiability scale. Apparently, respondents find it easier to justify research when it is associated with specific purposes rather than specific animal species, perhaps because purpose highlights the potential benefits of the use whereas the species highlights the typical harms to the subject being used. This difference is magnified if we compare species scales to purpose scales that do not include animal production, chemicals, or cosmetics, three purposes deemed least acceptable in our study. With this change, mean purpose-based justifiability rises to 3.8 ± 0.9 for students and 4.0 ± 0.8 for faculty, compared to 3.1 ± 1.0 and 3.5 ± 0,8, respectively, for species.

Most animal research is conducted to gather biological, medical, or toxicological information relevant to humans. The utility of these studies depends on their translatability, or ability to teach us something about people, and this remains a controversial scientific topic [41–43]. Our final research question asked respondents to identify the extent to which they had confidence in translatability of animal data to humans. For this question, gender differences in belief in translatability were not significant. Highest scores were associated with faulty in the biological sciences and with students or faculty who had participated in an animal research project, and lowest scores for those identifying as vegetarians or vegans. As observed for justifiability of purpose and species, belief in translatability was strongly correlated with overall support for animal research.

### Academic discipline and animal research justifiability

Gallup and Beckstead [44] reported that biology students were more favorable toward human use of animals and biomedical research (36,37). We noticed this trend when comparing biology students to social sciences and arts and humanities students, although these differences were not significant.

In contrast, academic discipline had a marked correlation with the response of faculty to most of our research questions. Faculty in biological sciences were most comfortable with human use of animals, followed by physical sciences, social sciences, and finally arts and humanities. It is tempting to suggest that the views of biologists would be influenced by their greater familiarity with animals as biological organisms and the norms of their disciplinary colleagues who routinely use them as objects of study and research. Those in arts and humanities may be more familiar with philosophical, literary, artistic, or humanistic views about animals, with physical and social sciences in between.

Discipline-specific differences between student and faculty scores also are striking. In the physical sciences and arts and humanities, student and faculty scores were always very close. For biological and social sciences, they decreased for AAS-6 or increased for ARAS-P and ARAS-S from student to faculty. Thus, the overall student-faculty differences in scale scores are driven by biological and social sciences, with faculty in both disciplines reporting greater justifiability. Further, recall that male ARAS-P and ARAS-S scores are identical between students and faculty, but female faculty scores are significantly higher than female student scores. In sum, disciplinary differences among undergraduate student perspectives toward human use of animals, including in research, are not significant. Apparently, these perspectives remain unchanged between students and faculty within physical sciences or arts and humanities, and they change from student to faculty, primarily for women, in biology or social sciences in a direction more favorable toward animal use.

### Interacting factors underlie extent of support for animal research

Our findings allow us to consider several possible conclusions about our student and faculty respondents’ views on animals and animal research. We have discussed the influence of gender and discipline above. Next, when other variables are held constant, dietary preferences and animal research experience also have strong effects. Being a vegetarian or vegan is negatively correlated with justifiability, and experience with an animal research project is positively correlated, though with a smaller magnitude. Others [45,46] have reported much lower agreement with animal research among vegetarian/vegans compared to non-vegetarians, and higher agreement among animal researchers. Finally, an individual’s answers to our survey questions tend to reflect a consistent worldview about animal research, and animals in general. Our animal attitude scale and animal research attitude scales are highly correlated (inversely, reflecting the reverse directionality of questions about general attitude versus research attitudes). As noted, scores on all scales also correlate with animal-related life choices and experiences and with belief in animal research translatability to humans. Establishing causal links among these variables is difficult, but internal consistency is a hallmark of most strong human beliefs [47].

Even so, both populations, and most demographic subpopulations, shared certain beliefs. Some members of all groups could find some animal research objectives justifiable. Justifiability in the context of animal research objectives and species can be divided into two ethical components in our current regulatory environment: substantive and procedural. Our substantive ethical beliefs determine whether we view certain examples of animal research as justified, because they produce more benefits than harms (in a utilitarian or consequentialist ethical framework) [2–6], or because they are sufficiently important to overcome the threshold presumption against harming animals (in a deontological ethical framework) [48]. Procedural ethical considerations can be defined as how well the laws and regulations are implemented, adhered to, and enforced [4]. Our survey does not allow us to distinguish between these components of justifiability, and subpopulations may have been differentially influenced by these subcategories. In a previous publication about our survey results [12], we reported that many faculty and most students lacked confidence in their familiarity with rules and regulations governing animal research, and with how effectively they were enforced, suggesting that non-procedural ethical considerations may be the primary determinant of justifiability by most of our study participants.

### Consequences for communication and science policy development

Our findings specifically address key research questions proposed in “Developing a Collaborative agenda for Humanities and Social Scientific Research on Laboratory Animal Science and Welfare” [49]. That report calls for “new research in the humanities and social sciences to inform emerging discussions and priorities on the governance and practice of laboratory animal research, and our findings are particularly relevant to communication about animal research and to assessing the harm-benefit analyses that are conducted to determine whether any specific proposed use of research animals should be approved.

First, our data make it clear that broad, if conditional, support exists on campus for the use of animals in research. As discussed above, conditions include each study’s research purpose and animal species. Multivariate analyses indicated similar support by faculty and students except for ARAS-P, for which the slight decrease in support by students might be associated with their younger average age.

Second, we nevertheless must remember that animal research justifiability exists on a continuum, whether related to general views on animals, research objective, species employed, or belief in translatability. Because views are not uniform, we need to be willing to address questions and concerns at the level of our audiences, with relevant specifics rather than broad generalities. Public controversy typically erupts over very specific uses of animals and involves audiences who are neither unconditionally supportive nor unconditionally opposed to animal research. Responding to controversy only with broad statements will fail to address, and hence resolve, the disagreement. Moreover, we should not expect to convince all students and faculty that particular decisions to use animal are right or wrong. But we should be willing to explain, to any listener, why we consider our decisions and associated actions justified, while respecting their right to disagree. If we are not willing to publicly explain our decisions about animal research, then we should not act on them. We reported previously [12] that both students and faculty want to hear more information from the university about the issues raised by animal research. University administrators and spokespersons should take advantage of this fact and expand the development of communication designed to improve understanding and build a larger consensus on the controversies raised by animal research. This is especially important for students and faculty outside of the biological sciences.

Third, our findings can help direct public policy related to the use of animals in research. Public policy should reflect reasonable public concerns, and our data illustrates factors that our campus community considers to be important. In particular, differential support for specific research purposes and use of specific animal species should be incorporated explicitly into the animal research review and approval process. How, then, are research purpose and species embedded into our current regulations?

The U.S. Government Principles for the Utilization and Care of Vertebrate Animals Used in Testing, Research, and Training [50] identifies regulatory considerations that must be applied to the review and approval of all Public Health Service and National Science Foundation proposals that request use of vertebrate animals for research, teaching, or outreach. The Animal Welfare Act and associated Regulations, enforced by the United States Department of Agriculture [51], applies to the use of warm-blooded vertebrates, except for agricultural species used in food or fiber research and rats, mice, or birds bred specifically for research use. Various federal and scholarly scientific societies also address expected review criteria. All require that proposed animal use be preapproved by an Institutional Animal Care and Use Committee (IACUC), which must include a veterinarian trained to work with the species encountered, a scientist with expertise in experiment design, and at least one member not affiliated with the sponsoring institution to represent the general public; see [4,52].

In the aforementioned documents, guidelines for the review of animal activities are described in very general terms. Regarding research purpose, the Principles states “procedures involving animals should be designed and performed with due consideration of their relevance to human or animal health, the advancement of knowledge, or the good of society” [50]. IACUCs must decide whether each proposed use of animals meets these criteria, but the Principles are silent on how these criteria are to be interpreted or applied. Regarding species, the Principles states “the animals selected for a procedure should be of an appropriate species and quality.” Here, the Principles only address the suitably of the animal for the research purpose, an important consideration but one that is entirely distinct from whether the species should be accorded greater or lesser consideration because of the human-animal bond, their position on the evolutionary phylogenetic scale, or the range of cognitive and emotive capacities they possess.

IACUCs also are guided by the “3Rs”, or reduction, replacement, and refinement, first proposed by Russell and Birch in 1959 [53]. Tannenbaum and Bennett discussed how these are now incorporated into several regulatory and guidance documents [54]. Replacement was defined originally as substituting non-sentient material for sentient material; it now is interpreted variously to include substituting “less sentient” or “phylogenetically lower” organisms [54]. Unfortunately, there is no consensus on how to define sentience in a way that would permit comparisons between species in an ethically and empirically justified manner. While our survey results support the idea that phylogeny is an important characteristic for our community, different lines of evidence, such as morphological traits versus genetic traits, can support different hypotheses about phylogenetic relationships [55], and the ranking by many of the use of cats and dogs as more justifiable than the use of nonhuman primates indicates that phylogenetic considerations can be outweighed. Again, the Principles give no guidance on resolving such conflicts.

Finally, a recent proposal for refining the harm-benefit analysis used to guide approval also suggests considering the relative “societal concern” of a species [6]. Thus, while there are suggestions that species is a relevant review consideration independent of its appropriateness for addressing a specific research question, there is little guidance about how this consideration should be implemented.

The consistent findings from many public surveys tell us about the public’s concerns and preferences regarding animal use in research, and animal users should refine their application of this knowledge to decisions about whether specific research can be approved. As long-serving IACUC members and chairs, two of the authors (EPS, RS) recognize that animal research purpose and species are part of IACUC deliberations. However, the review process would be improved if their consideration was more explicit, quantitative, and informed by published guidelines developed through robust ethical and empirical methodology. Taking this approach can assure university community members and the public that actual practices are consistent with the underlying beliefs of the community that animal research is intended to serve. More generally, when public concerns about animal research are taken into account, resulting decisions are viewed as being more rational and morally as well as democratically legitimate [10].
